# Ion-Locking in Solid Polymer Electrolytes for Reconfigurable Gateless Lateral Graphene p-n Junctions

**DOI:** 10.3390/ma13051089

**Published:** 2020-03-01

**Authors:** Jierui Liang, Ke Xu, Swati Arora, Jennifer E. Laaser, Susan K. Fullerton-Shirey

**Affiliations:** 1 Department of Chemical and Petroleum Engineering, University of Pittsburgh, Pittsburgh, PA 15260, USA; jil185@pitt.edu; 2 Department of Chemistry, University of Pittsburgh, Pittsburgh, PA 15260, USA; SWA10@pitt.edu (S.A.); j.laaser@pitt.edu (J.E.L.); 3 Department of Electrical and Computer Engineering, University of Pittsburgh, Pittsburgh, PA 15260, USA

**Keywords:** p-n junction, graphene, ion doping, electric double layer, polymer electrolyte

## Abstract

A gateless lateral *p-n* junction with reconfigurability is demonstrated on graphene by ion-locking using solid polymer electrolytes. Ions in the electrolytes are used to configure electric-double-layers (EDLs) that induce *p*- and *n*-type regions in graphene. These EDLs are locked in place by two different electrolytes with distinct mechanisms: (1) a polyethylene oxide (PEO)-based electrolyte, PEO:CsClO_4_, is locked by thermal quenching (i.e., operating temperature < T_g_ (glass transition temperature)), and (2) a custom-synthesized, doubly-polymerizable ionic liquid (DPIL) is locked by thermally triggered polymerization that enables room temperature operation. Both approaches are gateless because only the source/drain terminals are required to create the junction, and both show two current minima in the backgated transfer measurements, which is a signature of a graphene *p-n* junction. The PEO:CsClO_4_ gated *p-n* junction is reconfigured to *n-p* by resetting the device at room temperature, reprogramming, and cooling to T < T_g_. These results show an alternate approach to locking EDLs on 2D devices and suggest a path forward to reconfigurable, gateless lateral *p-n* junctions with potential applications in polymorphic logic circuits.

## 1. Introduction

The *p-n* junction—one of the building blocks of electronics—is required for diodes, bipolar and tunnel transistors, among other devices [1,2,3]. While two-dimensional (2D) materials have received attention for their unique properties and potential applications in electronics and optoelectronics [4,5], engineering *p-n* junctions in these materials presents unique challenges, such as achieving precise doping control over short distances for lateral homojunctions [2,6,7]. Nonetheless, plenty of progress has been made to engineer permanent *p-n* junctions in 2D materials. For example, graphene diodes [8,9], photodetectors [10,11], and photovoltaic cells [12] have been demonstrated. Generally, junctions in graphene are created in three architectures: lateral homojunctions formed at the interface between two regions of dissimilar doping [6], vertical junctions where two graphene sheets with dissimilar doping are stacked [3], and heterostructure junctions where a different 2D material is grown adjacent to or stacked with graphene (creating either an in-plane or vertical junction) [13,14,15]. 

Among the three architectures, the lateral *p-n* homojunctions offer a simple design with potential for large-scale integration, it does not require in-plane heterostructure growth [16], and it avoids vertical stacking and flake transfer [17,18]. The most commonly used approaches to achieve lateral *p-n* junctions is electrostatic doping using split gates [8,19], substitutional doping [6] and chemical doping resulting from charge transfer from adsorbates [20]. Less common approaches include applying large, local electrical stress [21], and substrate engineering [1]. Although the *p-n* junctions created by split gates are reconfigurable, challenges remain including fabrication and parasitics [22]. While graphene and other 2D materials can be substitutionally or chemically doped over wide areas *p-* or *n-*type, and with a dopant concentration range of 10^10^ to 10^13^ [6,23,24,25,26,27], one challenge is creating abrupt *p-n* junctions. Moreover, both substitutional and chemical doping [20] are permanent, meaning that the junction cannot be reconfigured. 

Similar to metal gates, electric double layer (EDL) gating is an electrostatic approach; however, EDL gating involves positioning ions at the interface between an electrolyte and a 2D surface by field effect. The ions induce image charge in the channel, and can achieve doping densities > 10^13^ cm^−2^ because of the < nm charge separation distance between ions and image charges [28,29,30,31,32,33,34,35,36]. Because this approach does not rely on replacing atoms in the 2D crystal or transferring charge, this method avoids permanently changing the crystal structure and potential problems associated with doping defects. Moreover, EDL doping is adjustable—ions can be reconfigured by changing the applied field. For these reasons, polymer electrolytes and ionic liquids have been used extensively for reconfigurable doping of graphene and other 2D materials [29,35,37,38,39].

Although EDL gating is a highly effective method to control charge transport in 2D materials, a constant gate bias is required to hold the ions in place and avoid EDL dissipation. What would be useful is a “gateless” electrolyte wherein the ions could be placed in a *p-n* junction configuration and locked into position via a trigger until they can be unlocked later and reconfigured. Such a triggerable locking/unlocking mechanism that enables reconfigurability on demand could be useful in applications requiring polymorphic electronics. That is, applications in which a circuit implementing one type of operating mode can reconfigure itself on demand and activate new functions in response to a stimulus [40,41,42,43]. 

One approach to “lock-in” an EDL is to decrease ion mobility by decreasing the temperature of the device below the glass transition temperature (T_g_) of the polymer electrolyte. This approach has been used previously to lock-in *p-n* junctions in MoS_2_ [44], WSe_2_ [45], and MoTe_2_ [29]. We have also used the same approach for unipolar doping of graphene, but with a polymer electrolyte, poly (vinyl alcohol) and LiClO_4_ (PVA:LiClO_4_), for which T_g_ > room temperature [31]. More than 75% of the EDL was retained at room temperature after removing the field, and the device could be reconfigured by heating to T > T_g_. In addition, the concept of ion-locking to form “frozen junctions” has been adopted in light-emitting electrochemical cells (LECs) where the homojunction of an electroluminescent organic semiconductor is created by fixing the ion distribution in a polymer electrolyte by cooling or forming covalent bonds [46,47,48].

Here, we take two approaches to create and lock-in a *p-n* junction in graphene. First, we apply the thermal quenching approach to lock a *p-n* junction using polyethylene oxide and CsClO_4_ (PEO:CsClO_4_). Then, we extend the concept to room-temperature operation via a custom-synthesized, doubly-polymerizable ionic liquid (DPIL) where a *p-n* junction is locked by a thermally triggered chemical reaction. Both approaches are “gateless” because only the source/drain terminals are required to create the junction. After the ions are locked in place using both approaches, backgated transfer characteristics show two Dirac points—a signature of a *p-n* junction in graphene [20,23,49,50]. These results suggest that appropriate chemical tailoring of the electrolyte can open possibilities for reconfigurable doping with potential use as polymorphic logic gates.

## 2. Materials and Methods

### 2.1. Device Fabrication

**Figure 1:**
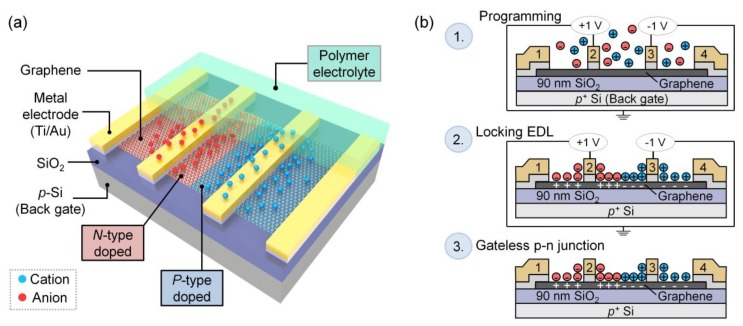
Schematics of a lateral graphene *p-n* junction with *n*- and *p*-type regions created by electric-double-layer (EDL) doping and subsequent ion-locking. (**a**) Four-electrode graphene device coated by a solid polymer electrolyte (i.e., PEO:CsClO_4_ electrolyte or DPIL). Cations (blue spheres) and anions (red spheres) dope the graphene channel to form *p*-type (pink) and *n*-type (blue) regions, respectively. (**b**) The formation of a *p-n* junction by EDL doping: (1) Programming voltages are applied to electrodes 2 and 3, while the remaining 2 electrodes and backgate are floated; mobile cations and anions redistribute in response to the field forming a *p-n* junction between electrodes 2 and 3. Note that the largest ion concentration occurs near the electrodes and decreases with distance away from the electrodes as modeled in reference [45]. (2) After the EDL is formed, the ions are locked into place either by cooling the PEO:CsClO_4_ device below the T_g_ of the electrolyte, or by DPIL polymerization. (3) After locking, the *p-n* junction remains in the absence of voltage.

The four-electrode graphene device used for this study is schematically illustrated in Figure 1a. The electrolyte was either PEO:CsClO_4_ or DPIL. Graphene flakes (~1.5 nm thick) were exfoliated from the bulk (2D Semiconductors) by mechanical cleaving (i.e., Scotch tape method) and transferred to a 1 × 1 cm^2^ p-type Si (Graphene Supermarket, resistivity 0.001–0.005 ohm⋅cm) with 90 nm SiO_2_. The substrate was pre-cleaned with acetone, isopropanol (IPA), deionized (DI) water and dried with N_2_. E-beam lithography (EBL, Raith e-LINE) was used to pattern the electrodes. PMMA-950-A4 (MicroChem) was spin-coated at 4000 rpm for 1 min and then annealed at 175 °C for 7 min. After exposure, the resist was developed in methyl isobutyl ketone MIBK:IPA (1:3 volume ratio) for 2 min, and rinsed with IPA for another 2 min. E-beam evaporation (Plassys Electron Beam Evaporator MEB550S) was used to deposit metal electrodes (3 nm Ti; 130 nm Au) at a base pressure < 1 × 10^−6^ Torr. Lift-off was performed in acetone overnight for 9 hours, followed by IPA, DI water rinse and N_2_ drying. Using contact-mode atomic force microscopy (AFM, Bruker Dimension Icon), e-beam resist residue was removed from the graphene between the electrodes (SCM-PIT-v2, 3 Nm^−1^) [33]. Before electrical measurements, samples were annealed at 127 °C (400 K) in vacuum at a pressure of 9 × 10^−7^ Torr for 4 hours. Electrical measurements were made on a Cascade Microtech Summit 11000 probe station (PEO:CsClO_4_ doped samples) or a Lakeshore cryogenic vacuum probe station (DPIL samples), both using a Keysight B1500A semiconductor parameter analyzer. After initial measurements on bare devices, the samples were transferred to an Ar-filled glovebox (H_2_O and O_2_ concentration < 0.1 parts-per-million (ppm)) for electrolyte deposition. 

### 2.2. Electrolyte Preparation

PEO:CsClO_4_ was prepared inside a glovebox as reported previously [29]. In brief, PEO (Polymer Standards Service, molecular weight 94,600 gmol^−1^) and CsClO_4_ (Sigma-Aldrich, St. Louis, USA, 99.9%) were dissolved in anhydrous acetonitrile (Sigma-Aldrich) to make a 1 wt % solution with the ether oxygen to Cs molar ratio 76:1. In the glovebox, the polymer electrolyte was drop-cast (25 µL over 1 cm^2^) onto bare graphene devices, dried at room temperature until the majority of the solvent evaporated, and then annealed at 80 °C for 3 min. The thickness of the cast electrolyte was reported previously as ∼1 µm [29]. The samples were transferred back to the probe station to form the *p-n* junctions. Programming voltages on electrodes 1 and 4 (V_1_ = +1 V; V_4_ = −1 V) were held for 10 min to drive ions into position, and the device was then cooled to 220 K which is below the T_g_ of PEO electrolyte (~ 242 K [29,51]) at a cooling rate of 0.7 K/min to immobilize ions and to fix the *p-n* junction with programming voltage still applied. The temperature was controlled by a Lakeshore 365 temperature controller to within ± 0.01 K. Once 220 K was reached, the sample was held at 5 min prior to the measurement to establish thermal equilibrium. After the measurement, the sample was heated to room temperature again where the *p-n* junction was reprogrammed to *n-p* by reversing the applied voltages (V_1_ = −1 V; V_4_ = +1 V).

DPIL monomers, 1-[(2-methacryloyloxy)ethyl]-3-methylimidazolium 1-[3-(methacryloyloxy) propylsulfonyl]-1-(trifluoromethane-sulfonyl)imide, were synthesized according to reference [52,53,54]. One mg of the initiator, azobisisobutyronitrile (AIBN), was dissolved in 100 μL unpolymerized DPIL monomer, and then drop-cast in the glovebox (25 µL over 1 cm^2^) with thickness estimated to be ~200 µm according to the cast volume. The samples were transferred to the probe station via a load-lock using an Ar-filled stainless steel suitcase. A programming voltage (V_2_ = +1 V and V_3_ = −1 V) was held for 10 min for *p-n* junction formation, followed by a 6-hour polymerization anneal at 353 K with the voltage applied, which is more than sufficient for immobilizing ions by polymerization (Figure S1). After polymerization, the device was cooled at ~ 0.3 K/min to room temperature and the programming voltage was removed. 

### 2.3. P-n Junction Formation

Schematics showing the process of *p-n* junction formation and ion-locking are shown in Figure 1b. At room temperature, the device is programmed by applying +1 V to electrode 2 and −1 V to electrode 3 (electrodes 1, 4 and the back gate are floated). Mobile ions in the electrolyte respond to the applied field (Figure 1b, row 1). Cationic or anionic EDLs form at the interface between the electrolyte and the graphene channel/metal electrodes. As modeled in reference [45] where ions are used to create *p-n* junctions, the highest concentration of ions (~2.4 × 10^21^ cm^−3^ at 1.5 V) builds up near the electrode surface and then dissipates with distance away from the electrode, meaning that a more abrupt junction can be expected with decreasing channel length. Note that the device channel lengths reported here are 100 times larger than those modeled in reference [45]; however, a channel length of tens of nanometers is not required to observe a junction, as demonstrated previously for MoTe_2_ [29] and MoS_2_ [44]. Thus, the junction formed in this study is either a *p-n* or *p-i-n* junction. To fix the *p-n* junction, ions are locked in place by either cooling the PEO:CsClO_4_ coated sample to T < T_g_ (where T_g_ ~ 242 K) or by polymerizing the DPIL at 353 K (Figure 1b, row 2). After locking, the voltages are removed and the *p-n* junction persists (Figure 1b, row 3). 

## 3. Results and Discussions

**Figure 2:**
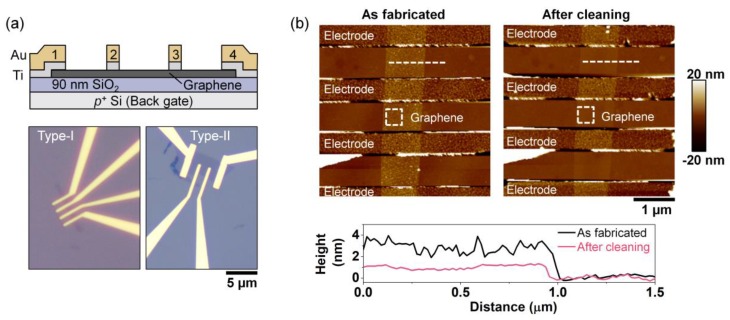
Bare, 4-electrode graphene devices. (**a**) Device schematic and optical images of two types of devices (electrode spacing ~ 1 µm). Type-I device has four electrodes with the same length; Type-II has two, shorter middle electrodes. (**b**) AFM topology scan of a representative graphene device as fabricated with e-beam resist residue (left) and after AFM cleaning (right). The white boxes (400 × 400 nm) on the AFM scans indicate one of the six locations over which roughness was measured; the dashed lines indicate the locations of the line scans and the corresponding data are shown below the AFM scans.

A cross-sectional schematic and optical images for two types of backgated, 4-electrode graphene devices are shown in Figure 2a. The type-I device has 4, equivalently sized electrodes where the voltage to create the junction is applied between the two inner electrodes (i.e., 2 and 3) separated by 1 µm. The type-II device also has four electrodes, but the two middle electrodes are shorter than the outer. In type-II, the outer electrodes (i.e., 1 and 4) are separated by 4.6 µm are used to create the junction, while the two middle electrodes are used to sense the junction. Note that these two geometries are not designed to work with a specific type of electrolyte—the *p-n* junction formation process should be similar for both. Because the *p-n* junction relies on EDL formation at the surface of graphene, AFM cleaning was used to remove residue from the EBL resist, thus enabling the ions to be positioned at the closest possible distance from the channel to maximize the capacitance density [33]. Figure 2b shows the topology of the channel surface for one representative graphene device measured before (left, as fabricated) and after (right) AFM cleaning. Line scans at the same location before and after cleaning indicate a channel thickness of ~1.2 nm (four layers) and ~2.2 nm of removed residue. The roughness of the channel surface was reduced from 1.32 ± 0.14 nm to 0.23 ± 0.02 nm after AFM cleaning, which is close to the surface roughness for freshly cleaved graphene on SiO_2_ [36,51,55]. Note that the maximum current and mobility are not degraded after AFM cleaning - in accordance with our prior reports [33,56].

### 3.1. P-n Junction Formation Using PEO:CsClO_4_

**Figure 3:**
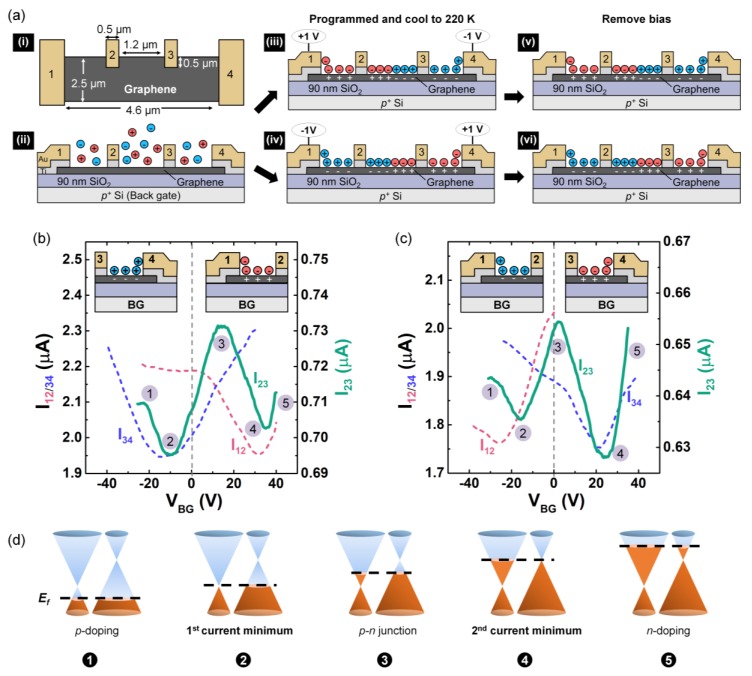
Using a polymer electrolyte (PEO:CsClO_4_) to create a lateral *p-n* or *n-p* junction in graphene. (**a**) Schematics of (i) device top view and (ii) device with homogeneously distributed ions before programming. Equal and opposite voltages are applied to electrodes 1 and 4 ((iii) V_1_ = +1 V and V_4_ = −1 V; (iv) V_1_ = −1 V and V_4_ = +1 V) to program the device, followed by cooling to 220 K to lock ions while the voltage is applied. Note that electrodes 2, 3 and the back gate are floated during programming and ion-locking. After locking, (v) *p-n* or (vi) *n-p* junctions are fixed in the absence of an applied field. (**b**) Backgated transfer characteristics (T = 220 K) corresponding to the doping profile in (iii), with V_D_ = 10 mV and a sweep rate of 3 V/s. Single current minima correspond to *p*- and *n*-type doping between electrodes 1-2 (red dash) and 3-4 (blue dash), respectively. Two current minima correspond to the *p-n* junction between electrodes 2-3 (green solid line). (**c**) Transfer characteristics corresponding to the to the doping profile in (iv). (**d**) Fermi-level tuning that gives rise to the I_23_-V_BG_ transfer characteristics in (**b**) and (**c**). The left and right cones represent the *n*- and *p*-type regions in the absence of V_BG_ after ion-locking, respectively.

PEO:CsClO_4_ has a low gate-to-source leakage current [57] and can induce charge carrier densities exceeding 10^13^ cm^−2^ in 2D materials [29,30,32,37]. In addition, Cs^+^ has a larger ionic radius than Li^+^ and is therefore less likely to undergo intercalation, as reported for LiClO_4_ [58,59]. While we have used it previously to induce a *p-n* junction in MoTe_2_ with Type I device geometry [29], here, we used it to create a *p-n* junction in graphene, and with a Type-II geometry. Schematics showing the mechanism are provided in Figure 3a, following the same general procedure as described in Figure 1. Voltage was applied to electrodes 1 and 4 (with electrodes 2, 3 and the back gate floated) at room temperature where the ions were mobile (T_g_ ~ 242 K) [51] to create the *p-n* junction. While continuing to apply the voltages, the device was cooled to 220 K (i.e., T < T_g_), which was sufficient to immobilize the ions and lock the EDLs [29,31]. After locking, the *p-n* junction forming voltages were no longer applied and the electrical characteristics were measured. After the measurements, the device was heated to room temperature, the voltage polarities were reversed to create an *n-p* junction, and the device was cooled again and measured. 

The transfer curves for the *p-n* and *n-p* configurations are shown in Figure 3b,c. The current between electrodes 1–2 (I_12_, red dash) exhibited a single current minimum at V_Dirac_ = +32 V in (b) and V_Dirac_ = −25 V in (c) correspondent with *p*-type doping by anions (ClO_4_^-^), and *n*-type doping by cations. Similar, but opposite observations were made for current measured between electrodes 3–4. Focusing now on the current between electrodes 2–3 where the *p-n* or *n-p* junctions exist, rectifying behavior was not expected in graphene due to the lack of a bandgap. Instead, the signature of the junction in graphene was a double current minimum in the transfer characteristics [20,23,49,50]. Here, the minima in both configurations occurred at voltages similar to those in the unipolar doped cases of electrodes 1–2 and 3–4. These data can be understood considering the band structure and Fermi level tuning of graphene with *p-* and *n-*doped regions as illustrated in Figure 3d. The first current minimum (at negative V_BG_) occurred when the Fermi level aligned with the Dirac point of the *n*-type graphene doped by cations after locking. Similarly, the second current minimum (at positive V_BG_) occurred when the Fermi level aligned with the Dirac point of the *p*-type graphene. 

### 3.2. P-n Junction Formation Using DPIL

**Figure 4:**
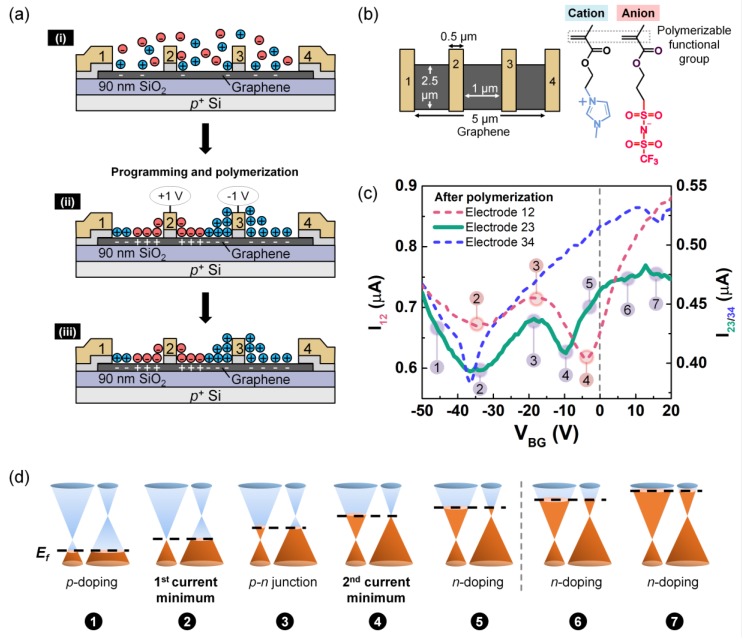
Using DPIL to create lateral *p-n* junction. (**a**) Schematics of (i) device with DPIL before programming; (ii) equal and opposite voltages are applied to electrodes 2 and 3 (V_2_ = +1 V and V_3_ = −1 V) while electrodes 1, 4, and the back gate are floated. After programming, the polymerization is thermally triggered at 353 K to lock the ions in place with the voltages applied; (iii) voltages removed after locking and cooling to room temperature, resulting in a fixed *p-n* junction. (**b**) Top view schematic and chemical structure of DPIL monomers. (**c**) Backgated transfer characteristics after polymerization between electrodes 1–2 (red dash), 2–3 (green solid line) and 3–4 (blue dash), with V_D_ = 10 mV and a sweep rate of 0.2 V/s. (**d**) Fermi-level tuning that gives rise to the I_23_-V_BG_ and I_12_-V_BG_ transfer characteristics after ion-locking.

While ion-locking at low temperature proves that stable junctions can be created in graphene by immobilizing ions, this low-temperature approach is impractical. However, there are other methods by which to immobilize ions for room temperature operation, and we explored a chemical trigger here. DPIL has cations and anions with similar structure as the ionic liquid, [EMIM][TFSI] (1-ethyl-3-methylimidazolium bis(trifluoromethylsulfonyl)imide), which has been successfully used as an ion gate [38,60], but with the modification of polymerizable functional groups (carbon-carbon double bonds in methacrylate, Figure 4b) on both charged species to perform ion-locking. Before polymerization, DPIL monomers behaved as a typical ionic liquid with mobile ions that can form EDLs in response to an applied field, Figure 4a. Once the ions were in place, heat was used to trigger DPIL polymerization, which immobilized the ions [54,61]. After polymerization, the programming voltages were removed, the device was cooled to room temperature, and transfer characteristics were made (Figure 4c).

One of the most obvious differences between the data in Figure 3 and Figure 4 is that the current in the DPIL-gated devices in Figure 4 was modulated using a negative V_BG_, which means that the DPIL was causing an overall *n*-type doping of the graphene channel. Note that such an *n*-type shift was not observed on the bare graphene device (Figure S2). However, this result is not particularly surprising when considering the molecular structure of the cations and anions. The charged functional group of the cation (1-ethyl-3-methylimidazolium (EMIM)) had π orbitals that showed a stronger affinity to graphene due to π-π interactions compared with the charged functional group of the anion (bis(trifluoromethylsulfonyl) (TFSI)) [62]. This structural distinction, combined with the longer polymerizable group on the anion monomer, suggests that DPIL cations were more likely than anions to be located at the graphene surface. The stronger chemical affinity of the cations and their lower steric restrictions would lead to overall *n*-type doping. This preference for cations to be located near the surface is emphasized schematically in Figure 4a.

Focusing again on Figure 4c, I_34_ showed a current minimum at V_BG_ = −37 V, which is consistent with the unipolar *n*-type doping that would be expected between electrodes 3 and 4, in accordance with the schematic Figure 4 (a, iii). As with PEO:CsClO_4_, I_23_ also showed double current minima (1^st^ V_Dirac_ = ~ −35 V; 2^nd^ V_Dirac_ = −10 V), the signature of a *p-n* junction and an indication that the ions were locked into place. However, unlike the PEO:CsClO_4_-gated device, I_12_ also exhibited two current minima (1^st^ V_Dirac_ = −33 V; 2^nd^ V_Dirac_ = −4 V). At first blush, a *p-n* junction would not be expected between electrodes 1 and 2. However, as discussed above, the cations of the DPIL seemed to have affinity for the graphene surface, which means that they would also preferentially accumulate near electrode 1, which was floated during programming and locking. Thus, the preferential accumulation of cations to the graphene surface can explain the additional *p-n* junction located between electrodes 1 and 2. In the future, the *p-n* junction between electrodes 1 and 2 can be avoided by further material optimization to avoid preferential adsorption of one type of ion over the other. The corresponding band diagrams are shown in Figure 4d with a similar interpretation as shown in Figure 3d for PEO:CsClO_4_, except for the prominent *n*-type shift observed for DPIL. 

The results of the chemically locked, gateless, graphene *p-n* junction serve as proof-of-concept that alternate triggers can be used to semi-permanently dope 2D devices for room temperature operation. In fact, we have also recently shown that incorporation of thermally-labile linkers into polymerizable ionic liquids allows ions to be released after the materials are polymerized, which we anticipate will enable DPILs that can be locked and then unlocked with a second trigger [54]. This type of locking and unlocking—especially if it can be field-controlled—could prove useful for polymorphic circuits. Moving forward, some of the challenges include reducing the heavy *n*-type doping caused by DPIL polymerization, locking and unlocking the EDLs on demand to demonstrate reconfigurability, and extending the concept to 2D semiconductors to show rectifying behavior. 

## 4. Conclusions

A gateless lateral *p-n* junction is demonstrated on 4-electrode graphene devices by ion-locking using two different solid polymer electrolytes. PEO:CsClO_4_ is locked by lowering the operating temperature to 220 K (i.e., below its T_g_), while DPIL is locked by heating to 353 K to polymerize the anions and cations, and then operating the device at room temperature. In both cases, the signature of the *p-n* junction is double current minima—consistent with what is expected for graphene. For the PEO:CsClO_4_, reconfigurability of the *p-n* junction is also demonstrated by resetting the device at room temperature, reversing the polarity of the applied bias, and cooling the temperature to 220 K to create a *n-p* junction. While the chemical trigger using DPIL is more practical than the low-temperature approach, preferential *n*-type doping is observed and attributed to the affinity of the cation over the anion for the graphene surface. Overall, this study provides proof-of-concept that triggerable electrolytes can lock-in *p-n* junctions in graphene, and this approach can be extended to other 2D material, for electronic and optoelectronic applications. 

## Supplementary Materials

The following are available online at https://www.mdpi.com/1996-1944/13/5/1089/s1, Figure S1: Change of ion mobility after polymerization, Figure S2: Transfer characteristics of as-fabricated device.
