# Supplementary material for: Ion-Locking in Solid Polymer Electrolytes for Reconfigurable Gateless Lateral Graphene p-n Junctions
Source: Materials (Basel). 2020 Mar 1;13(5):1089. doi: 10.3390/ma13051089 (PMC7084918; doi:10.3390/ma13051089)
Supplement: Supplementary file 1 [file materials-13-01089-s001.pdf]

materials

Supporting Information

Ion-Locking in Solid Polymer Electrolytes for Reconfigurable Gateless Lateral Graphene p-n Junctions

Jierui Liang [1], Ke Xu [1,*], Swati Arora [2], Jennifer E. Laaser [2] and Susan K. Fullerton-Shirey [1,3,*]

[1] Department of Chemical and Petroleum Engineering, University of Pittsburgh, Pittsburgh, Pennsylvania 15260, USA; jil185@pitt.edu
[2] Department of Chemistry, University of Pittsburgh, Pittsburgh, Pennsylvania 15260, USA; SWA10@pitt.edu (S.A.); j.laaser@pitt.edu (J.E.L.)
[3] Department of Electrical and Computer Engineering, University of Pittsburgh, Pittsburgh, Pennsylvania 15260, USA
* Correspondence: fullerton@pitt.edu (S.K.F.-S.); ke.xu@pitt.edu (K.X.)

Received: 1 December 2019; Accepted: 28 January 2020; Published: date

I. Change of ion mobility after polymerization

The changes in the mobile ion content of the DPIL before and after polymerization was assessed by depositing DPIL across two parallel metal electrodes in a capacitor geometry. The metal electrodes (Ti/Au, 3/140 nm) were patterned on an Si/SiO_2 substrate with dimensions 20 (width) × 200 μm (length) for each electrode, and a separation distance of 20 μm. The preparation and deposition of DPIL with thermal initiator were the same as described in section 2.2 in the manuscript. The annealing time for polymerization was set to be 1 hour followed by the cooling to 298 K. The charging current was measured on one electrode where a +1 V step voltage was applied. The other electrode was grounded, and the back gate was floated throughout the measurement. Note that the first data point was recorded at ~ 2 ms which is longer than the timescale for dielectric charging [1], and therefore the data shown here are purely an ionic response.

Before polymerization, the charging current exhibits an initial spike as the mobile ions in the DPIL begin to migrate toward the electrodes; this current decays to zero as the ions reach their new equilibrium distribution under the electric field. In contrast, no charging current is detectable after 1 hour of thermal polymerization, indicating no detectable mobile ions in DPIL. The result indicates that the annealing of 1 hour at 353 K is sufficient to immobilize ions by polymerization.

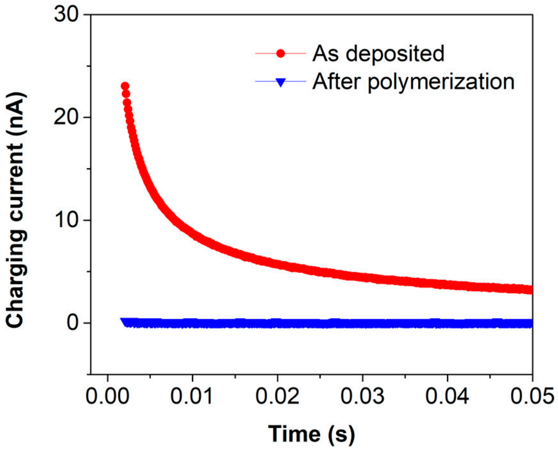

Figure S1. Capacitor charging of DPIL at 298 K, measured as freshly deposited before polymerization (red curve) and after a 1-hour thermal polymerization at 353 K and cooling back (blue line). The two metal electrodes (Ti/Au, 3/140 nm) are separated by 20 μm. At time = 0, a step voltage of +1 V was applied on one electrode where the current was monitored, while the other electrode was grounded.

II. Transfer characteristics of as-fabricated device

Backgated transfer characteristics were made on the bare graphene device (the same device in Figure 4) in a vacuum probe station using a Keysight B1500A semiconductor parameter analyzer. The results show that the bare graphene device has a single current minima near zero between all electrodes.

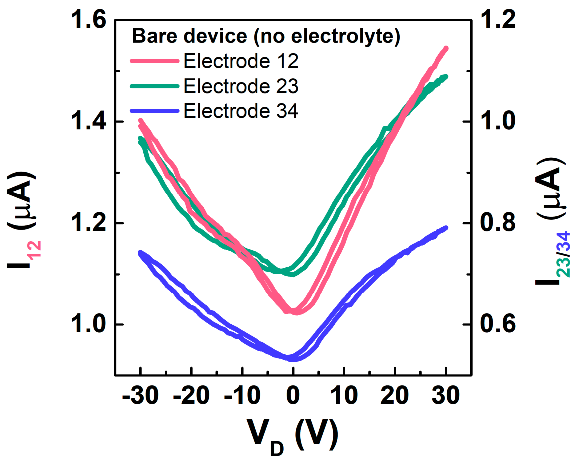

Figure S2. Backgate transfer characteristics of a bare graphene device, with V_{DS} = 10 mV and a sweep rate of 3 V/s.

References

1. Xu, K.; Islam, M. M.; Guzman, D. M.; Seaubaugh, A.; Strachan, A.; Fullerton-Shirey, S. K. Pulse Dynamics of Electric Double Layer Formation on All-Solid-State Graphene Field-Effect Transistors. *ACS Appl. Mater. Interfaces* **2018**, *10*, 43166–43176.
